# Operational Parameters for the Aerial Release of Sterile Codling Moths Using an Uncrewed Aircraft System

**DOI:** 10.3390/insects12020159

**Published:** 2021-02-13

**Authors:** Evan D. Esch, Rachael M. Horner, Dustin C. Krompetz, Nathan Moses-Gonzales, Melissa R. Tesche, David Maxwell Suckling

**Affiliations:** 1 Okanagan-Kootenay Sterile Insect Release Board, Kelowna, BC VIW 3Z4, Canada; Mtesche@oksir.org; 2 School of Engineering, University of British Columbia, Okanagan Campus, Kelowna, BC V1V 1V7, Canada; 3 The New Zealand Institute for Plant and Food Research Limited, 8140 Christchurch, New Zealand; rachael.horner@plantandfood.co.nz (R.M.H.); max.suckling@plantandfood.co.nz (D.M.S.); 4 M3 Consulting Group Ltd., Dayton, OH 45068, USA; dustin.krompetz@M3cg.us (D.C.K.); nmosego@plantandfood.co.nz (N.M.-G.); 5 School of Biological Sciences, University of Auckland, 1072 Auckland, New Zealand

**Keywords:** sterile insect technique, *Cydia pomonella*, release method, uncrewed aircraft system, codling moth, drone, apple, pest management

## Abstract

**Simple Summary:**

Pest management through the release of sterile codling moths from drones or uncrewed aerial vehicles (UAVs) represents an efficient method for distribution over orchards. In this study, moths released from greater altitudes were more broadly distributed and drifted more in strong winds compared to those released from lower altitudes. Most of the released insects were recaptured in a 50 m wide swath under the release route. Recapture rates from aerially released insects were 40–70% higher compared to those released from the ground. We found that an uncrewed aircraft system (UAS) releasing insects from 35 m above the ground in release routes 50 m apart outperformed standard ground release methods. UASs provide a promising alternative to ground release and conventional aircraft for the release of sterile codling moths.

**Abstract:**

The codling moth is a serious pest of apples in most regions of the world where this fruit is produced. The sterile insect technique is one strategy used to control this pest and is employed as part of an area-wide integrated pest management program for the codling moth in British Columbia, Canada. Modified fixed wing aircraft are the most common method for the release of sterile insects in large area-wide pest management programs. However, aerial release with a full-size aircraft can be prohibitively expensive. We evaluated the use of small, uncrewed aircraft systems (UASs) for the release of sterile codling moths. Sterile codling moths released from greater altitudes were more broadly distributed and drifted more in strong winds, compared to those released from lower altitudes. Most of the released insects were recaptured in a 50 m wide swath under the release route. Recapture rates for aerially released insects were 40–70% higher compared to those released from the ground. UASs provide a promising alternative to ground release and conventional aircraft for the release of sterile codling moths.

## 1. Introduction

Apples are among the most abundantly produced fruits all over the world, with upwards of 50 Mt produced annually [1]. The codling moth *Cydia pomonella* (L.) is a key pest of apples in most regions of the world where this fruit is grown [2], including the valleys of the Southern Interior of British Columbia (BC), Canada [3]. This pest had established itself as a serious pest in the Okanagan and surrounding valleys by 1916, at which time its populations were managed with lead arsenate insecticide [4]. Lead arsenate was later replaced by organochlorine insecticides, and while the economic losses from codling moths were significantly reduced, the environmental and health hazards of these insecticides became too large to bear [4,5]. The desires to minimize the negative impacts of pesticide usage and slow the development of insecticide resistance promoted the development of alternate strategies for suppressing insect pests. One such autocidal approach to pest management, the sterile insect release method and its derivative inherited sterility (IS) (collectively referred to here as the sterile insect technique (SIT)), became widely known through its successful application in the eradication of the New World screw worm *Cochliomyia hominivorax* (Coquerel) [6]. While numerous researchers worked towards the development of SIT to control the codling moth, it was ultimately a 15-year research program by Proverbs and colleagues that led to the establishment of the Okanagan–Kootenay Sterile Insect Release (OKSIR) program [7]. 

**Figure 1:**
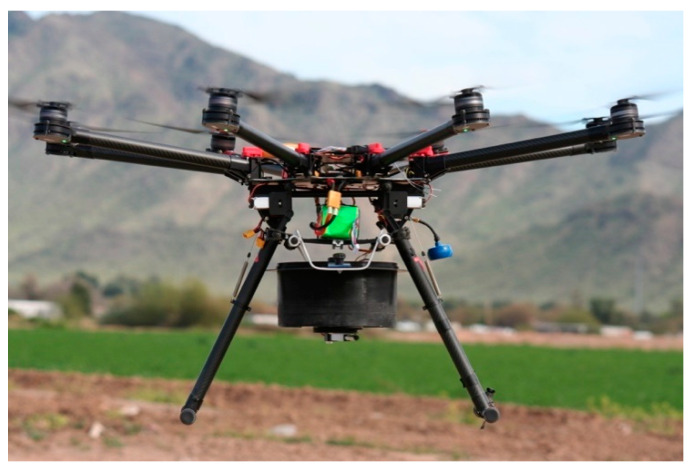
Photograph of the Hermes uncrewed aircraft system (UAS) used to release sterile codling moths in the Okanagan in 2016 and 2017. The UAS is shown in flight. The moth storage and release device is mounted under the airframe, between the landing gear.

The OKSIR program was established in 1992 with the stated goal of eradicating the codling moth from the Okanagan and surrounding valleys [7,8]. While eradication was never achieved, this program has conducted an effective, area-wide integrated pest management program (AW IPM), with SIT as the key component for more than 25 years [7,9]. During this time, research was conducted to advance the field of the codling moth SIT and promote its uptake globally [2]. Most of this research focused on optimizing the radiation dose to leverage IS [10,11,12,13], improving rearing methods [14,15,16], and integrating SIT with other pest management strategies [17]. Other research has served to identify limitations in the OKSIR program’s operations [18,19,20] or evaluate the global expansion of codling moth SIT [21,22,23]. Until a recent cooperative research program hosted by the International Atomic Energy Agency, there has been a comparative dearth of research evaluating release methods for sterile codling moth SIT [23]. Since the establishment of the cooperative program in 2016, uncrewed, fixed, and rotary-winged aircraft have been used operationally for five years in a test of over several hundred hectares of apple orchards in New Zealand using insects supplied by OKSIR [23]. Similarly, uncrewed rotary-wing aircraft (Figure 1) have been used to release sterile codling moths over an area of 1000 ha in experimental and commercial applications in Washington State, USA.

The sterile insect technique works by reducing the frequency of mating amongst the wild pest population and generally achieves this by outnumbering wild males with sterile ones [6]. Sexual competition between male codling moths can be described as a form of scramble competition [24,25]. However, the underlying codling moth populations are aggregated across different spatial and temporal scales [26,27], and the locations of these aggregations may be unknown to pest managers, making the goal of outnumbering the wild pest more challenging. As such, the OKSIR program has employed a strategy of uniformly distributing sterile insects within a given orchard [28] with varying release rates used between orchards according to their underlying wild populations [29]. This strategy is a means of “hedging bets” against the imperfect understanding of aggregations of wild codling moths. While the quality and competitiveness of sterile insects has been the focus of most of the research on the codling moth SIT, it is evident that the release methods and strategy also play an important role [30].

**Figure 2:**
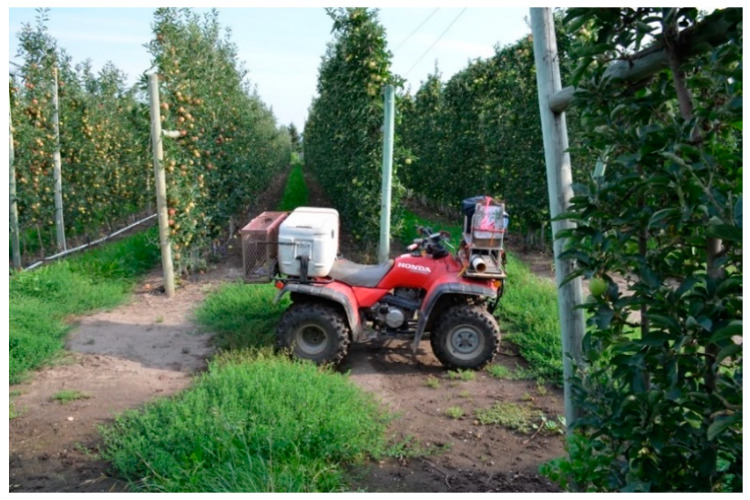
Photograph of a modified Honda Fourtrax all-terrain vehicle (ATV) used by the Okanagan–Kootenay Sterile Insect Release Program to release sterile codling moths (*Cydia pomonella*) in apple orchards. Moths are stored in a cooler on the rear of the ATV and released from the release device on the front of the vehicle (photograph by Tom Walker, 2015).

Aerial release with crewed, fixed-wing aircraft is the preferred method for most large AW IPM programs utilizing SIT [30]. Aerial release from crewed, fixed-wing aircraft rapidly delivers insects with excellent fitness in a uniform distribution. However, high fuel, pilot, and hangar costs make this a very expensive method, often amounting to more than a quarter of the operating budget of SIT programs [30]. Manned aircraft are also not suitable for release in a fragmented landscape where a more targeted release is required [30]. The OKSIR program explored the use of a crewed, fixed-wing aircraft (Cessna 205) to release sterile insects in 2003, but this method was fraught with logistical challenges (OKSIR, unpublished data). Rotary-wing aircraft were used for the aerial release of sterile codling moths early in the development of the OKSIR program, but this approach was ultimately deemed too expensive [8,31]. Instead, the OKSIR program has used modified all-terrain vehicles (ATVs) (Figure 2) to conduct moving, ground releases of sterile insects since it started releases in 1994 [28]. However, this procedure exposes the chilled sterile insects to significant stress during the transport and release process [18]. It has been argued [18] that the negative impacts of transportation and cold storage needed for ground release outweigh the numerous improvements to the codling moth SIT methods that have been developed in the last 25 years. Ground release methods also require a substantial amount of time and staff, and release vehicles are often restricted to roads or orchard rows, resulting in a less uniform distribution of insects [30]. The growing use of uncrewed aircraft systems (UASs) in agricultural applications present opportunities to deploy aerial release technology in situations in which the costs were previously prohibitive [32]. As part of the International Atomic Energy Agency Cooperative Research Program on “Improved Field Performance of Sterile Male Lepidoptera to Ensure Success in SIT Programs”, our Canadian, US, and New Zealand team conducted experiments to explore basic operational parameters to determine how a UAS might be used to release sterile codling moths in the context of the OKSIR program and to establish more universally useful guidelines for other programs. Aerial releases with UASs were evaluated against the standard ground release methods used by the OKSIR program. Given the widely accepted superiority of aerial release methods and the increasing use of UASs in agriculture, we hypothesized that aerial release from a UAS would be more effective than the ground release methods currently employed by OKSIR.

## 2. Materials and Methods

### 2.1. Rearing and Release

Moths used in these experiments were reared at the Okanagan–Kootenay Sterile Insect Release Program’s mass-rearing facility in Osoyoos, British Columbia, Canada, and prepared to the same standards as those used in program operations. Moths developed in trays (45 cm × 29 cm × 2.5 cm) on a modified Brinton diet under environmental control conditions [33]. Upon emergence, adult moths of both sexes were collected, immobilized by chilling to 2–4 °C, and packaged in 10 cm Petri dishes, with each dish containing ca. 800 moths in a 50:50 (M:F) sex ratio [14]. Petri dishes of moths were irradiated with a dose of 200 Gy in a Co^60^ irradiator (JL Shepherd and Associates, San Fernando, CA, USA), transferred to 2 L plastic bowls for marking, and then returned to Petri dishes for storage and transportation. Fluorescent powders from Dayglo Corp. (Cleveland, OH, USA) were used to mark the moths. Different colors of Dayglo powder were used for different release methods and replicates, allowing the identification of recaptured moths to be assigned to a given release. Marking was accomplished by sprinkling a half scoop (Hayman-Style Microspatulas, Fisherbrand^TM^) of fluorescent powder (ca. 0.05–0.07 g) over ca. 800 moths. Bowls were gently turned to ensure thorough coverage of moths with fluorescent powder. Efficacy of marking was evaluated under ultraviolet light at the start of each experiment (Figure S1). Moths were kept chilled at 2–4 °C at the rearing facility (Osoyoos, BC, Canada) or at satellite field offices (Kelowna, BC, Canada) until transportation to the field in a portable cooler. Moths were transferred from Petri dishes to the release devices immediately before release. 

The Hermes UAS was used for the aerial release experiments outlined below (Figure 1). This UAS has a combination of commercial and custom components, designed and built by M3 Consulting Group Inc. (Dayton, OH, USA). The UAS can be described as an “octocopter”, capable of vertical take-off and landing, with a moth storage and release device mounted under the airframe. The release of moths was monitored during each flight with a fish-eye lens and a digital transmitter in 2016. A significant redesign of the release mechanism occurred between 2016 and 2017 to improve the uniformity of moths released per unit of time. In 2016 and 2017, numerous bench-tests and flight-tests were conducted to calibrate the release rate, so that the release rate of moths per unit of time, the flight speed of UAS, and the spacing of flight paths could be manipulated to achieve the desired release rate of moths per area. Flight plans for the test releases were created based on geo-referenced orchard maps created by ArcMap and programmed in Mission Planner, ArduPilot [34].

The ground release of moths was performed using a Honda Fourtrax all-terrain vehicle (ATV) equipped with a custom release device and cooler for moth storage (Figure S2). The release device used a constant stream of air to gently propel moths from the device to the side of the direction of ATV travel, thereby releasing them onto the ground and the lower canopy of trees (after Proverbs) [28]. The speed of the travel (4–6 m/s on average) and release rate of moths were manually controlled by the ATV operator to achieve the desired number of moths per ha. Release routes were laid with flagging tape prior to all experiments and were followed by the ATV operator during releases. 

### 2.2. Experiment 1: Effect of Release Height on Moth Distribution, Drift, and Recapture

To better understand how aerial release distributes sterile moths in an orchard, a mark–release–recapture experiment was conducted on 17–19 August 2016. The test orchard selected for this experiment was a flat, 275 m × 250 m section of a larger apple orchard located in the Similkameen Valley, BC, Canada (49.145925°, −119.731873°). Predominantly Gala and Mcintosh varieties were planted in trellised, 3 m-wide rows, with 2.5–3 m-tall trees spaced 0.3–1 m apart in each row, creating a relatively uniform canopy. The test orchard was bordered by similar orchards to the north and south, with a small amount of similar orchards to the west, with a semi-natural area beyond, and a road to the east, with similar orchards beyond. The test orchard was divided into two equal size replicates, referred to as east and west plots hereafter, each 250 m × 125 m. There was a 25 × 250 strip between the edges of the east and west plots.

Multiple releases were conducted over the study orchard. Release routes were designated 50 m from the west edge of each plot, parallel to the long edge of the plot and proceeded from the south to the north end of the test orchard. A “release” consisted of the UAS taking off from a staging area near the test orchard, proceeding to the south end of the plot where the release route began, making a single pass along the release route from the south to north end of the plot while releasing moths, and then returning to the staging area. The UAS travelled at 3 m/s (ground speed). One released at 25 m above ground and one release at 50 m above ground were made in each plot, along the same release route, each day. A total of four releases were conducted each day. Releases were repeated on 3 consecutive days for a total of 12 releases or six replicates at each altitude. Flights commenced at 3:00 p.m., after all orchard workers left the area. Moths released from different altitudes and on different days were marked with different colors of fluorescent powder, for a total of 3 different colors. Temperature, relative humidity, wind speed, and direction were recorded before each flight with an AR816 handheld anemometer (Kestrel). The release device was calibrated to release 2000 insects/ha both on a lab bench and in flight. However, these calibrations did not account for the strong winds encountered during the test releases, and the device used in 2016 did not perform as expected, resulting in some moths left in the release device after flights. All of the unreleased moths were collected from the release device and weighed to estimate the actual number of moths released (OKSIR, unpublished data). A determination of the number of released moths was necessary to calculate the percent recapture.

White Delta II traps (Trécé Inc., Adair, OK, USA.) were used to recapture marked male moths in the test orchard. Traps were hung in the top third of the canopy in a 25 m × 25 m grid of 132 traps (ca. 7 traps/ha). Traps were baited with 1 mg (1×) codlemone sex pheromone lures (Trécé Inc., Adair, OK, USA). For Experiment 1, sticky trap liners were collected and replaced every day for 7 days, starting 1 day after release. Liners were examined under UV light to determine how many marked moths from each release were collected on each day at each grid location. 

### 2.3. Experiment 2: Comparing Recapture Rates between Aerial and Ground Release Methods

To evaluate the effectiveness of a UAS for sterile moth release, test flights simulating the operation requirements of the OKSIR program on 16–17 August 2017 were conducted. In a single “mission”, the UAS released moths into one orchard block each of small (ca. 0.5 ha), medium (ca. 2–3 ha), and large (ca. 5–8 ha) sizes during a single flight (Figures S3–S6). Four different “missions” were flown, each treating a different small, medium and large orchard block. Two missions were flown each day. The UAS flew at 8 m/s (ground speed) 35 m above ground releasing ca. 2000 moths/ha along release routes spaced 50 m apart. During a test flight, the UAS would launch, travel to and from test orchards, and release insects along designated release routes above the test orchards. Each block in a test flight was 30–200 m from the next test block and situated amongst mixed-fruit orchards and residential dwellings. Test orchards were flat and had diverse apple varieties as well as varied tree spacings ranging from 3 m × 0.3–6 m apart. All test blocks were located within a 350 ha area of East Kelowna, BC.

Ground releases were conducted using an ATV, one to two hours following the aerial releases. The release rate (2000 moths/ha) was controlled manually by E.D.E. for all experiments. The ATV travelled at 4–6 m/s along release routes ca. 30 m apart. Release routes differed between aerial and ground release according to tree-row orientation, fences, buildings, and other obstacles which differently affected navigation in air or ground. Instead, the release routes are examples of how the two different methods are/could be utilized operationally. Moths released from the air or ground were marked with different colors of fluorescent powder. White delta traps with 1x lures were used to recapture marked, male moths in the test blocks. Traps were spaced on a 50 m × 50 m grid, or as close to this configuration as possible, given the irregular shape of some blocks. Traps were checked one, four, and seven days after release.

A similar, unreplicated comparison of aerial and ground release was conducted in the Similkameen Valley on 16 August 2016. Around 2000 insects/ha were released in a rectangular, 3.5 ha orchard. The UAS released moths along a central release route running the length of the orchard at 25 m above ground, while the ATV released the same number of insects along 3 evenly spaced routes running the length of the orchard. Moths from aerial and ground releases were marked with different colored fluorescent powders. Moths were collected in white delta traps placed on a 25 m × 25 m grid. In this experiment, traps were checked every day for seven days, and moth coloring was identified as described above.

### 2.4. Experiment 3: Comparing Release Strategies

Different aerial release strategies were compared. Metered releases of moths along evenly spaced release routes (swaths) were compared to the release of moths from a single stationary point 35 m over the center of the orchard (burst) (Figures S7–S10). This was accomplished by adding burst releases over the medium-sized plots in Experiment 2 in 2017. Burst releases were conducted at a rate of 2000 insects/ha immediately following metered releases. Moths from burst releases were marked differently than other aerial and ground releases. The traps were the same as those used for Experiment 2. Comparisons between burst and metered releases were replicated four times.

### 2.5. Data Analysis

Recapture rates for each trap were calculated, based on the estimated number of male moths released, for each day after release and the total trapping period since pheromone-baited traps only attract males. For Experiment 1, the recaptures in each row of 11 traps at given distances perpendicular to the release route (−50, −25, 0, 25, 50, and 75 m) were tabulated and totaled for each test flight. The influence of release height and the absolute value of trap distance (0, 25, 50, and 75 m) from the release route on total recaptures was examined by two-way analysis of variance (ANOVA). Total recaptures from all traps in swath and burst releases were compared with a paired *t*-test. The pattern of recapture (swath) was described by heat maps of percent recapture for each release altitude and day after release. Spatial autocorrelation of recaptured moths was described using Moran’s *I* spatial statistic, calculated using the spdep package in R (R Core Team, Vienna, Austria), using k = 8 nearest neighbors, except for Experiment 3, in which k = 4 neighbors was used to accommodate the smaller plot sizes. Moran’s *I* has been used widely in the study of insect spatial patterns [35,36]. Moran’s *I* index compares the abundance of moths in neighboring traps (k) to the mean deviation in moth abundance in all traps and produces an index value between −1 and 1, describing the overall spatial autocorrelation for an array of points. Values of *I* equal to −1 indicate a perfectly even distribution, 0 indicates a random distribution, and values of 1 indicate a perfectly aggregated distribution. A Monte Carlo procedure with 99 permutations was used to test whether the observed distribution of moth captures was significantly more clustered than random. Recapture rates from aerial and ground releases in each orchard size (Experiment 2) were compared using two-way ANOVA. Only large orchard blocks from Experiment 2 had enough trap locations to calculate robust values of Moran’s *I*. This index was calculated using the same method as described above (k = 8) for each release method in the large blocks. 

All experimental orchards relied on sterile insect releases as the main tactic for controlling wild codling moth populations. Pesticide applications may have been used to supplement SIR, however, no pesticides were applied during the course of these experiments. Mating disruption was not used in any experimental orchards.

## 3. Results

### 3.1. Effect of Release Height on Moth Distribution, Drift, and Recapture

**Figure 3:**
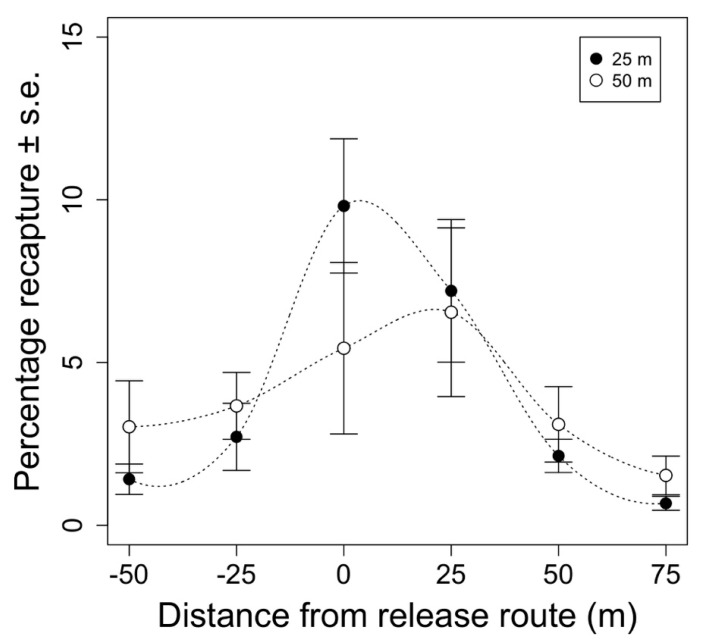
Mean percentage of recaptured moths vs. distance from the aerial release route (m). Each distance from the release route represents the sum of 11 traps in the grid at the specified distance from the release route for each replicate. Points represent week-long total recaptures averaged across replicates ±1 standard error for each distance (*n* = 6). Black and white points represent recaptures of moths that were release from 25 or 50 m above ground, respectively.

**Figure 4:**
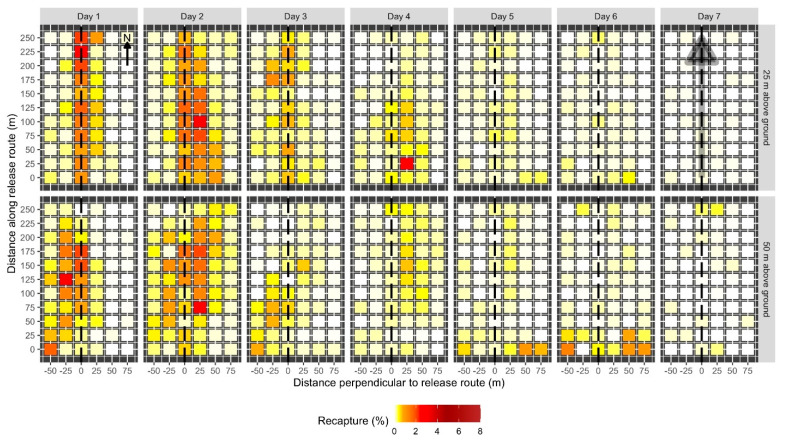
Spatial pattern of daily male codling moth recaptures when released from 25 m (top row) or 50 m (bottom row) above ground. Each day and altitude combination was replicated 6 times. Each colored cell represents the mean percentage of moths recaptured at a given location on a given day on a 25 m × 25 m grid of pheromone-baited traps. The intensity of shading in the cell corresponds to percentage recapture (see legend). The dashed line indicates the location of the release route over the plot. Locations of each trap along the plot and the distance perpendicular to the release route in m are indicated on the axes. Recaptures are scaled such that the sum of all cells for all seven days for a given release height equals 100%. The arrow in the upper left panel indicates plot orientation relative to north. Prevailing winds were out of the north. The shaded gray arrow in the top right panel indicates the location and direction of the release route.

Overall, 6542 marked moths were recaptured from the 12 test releases examining the effect of release height on moth distribution. Release height did not have a significant effect on the total proportion of recaptured moths (F_1,68_ = 0.014, *p* = 0.905). Total recaptures averaged 23 ± 2.1% (mean ±1 standard error) and 21 ± 2.0% when released from 25 and 50 m above ground, respectively. Most of the moths were recaptured directly under the release route our immediately next to it, with the proportion of recaptured moths decreasing as the distance from the release route increased (F_3,68_ = 21.880, *p* < 0.005). After seven days, 80% of the moths were recaptured in a 50 m wide swath under the release route when released from 25 m above ground. When released from 50 m above ground, 67% of the moths were recaptured in the same 50 m wide swath. While more moths tended to be caught farther away from the release route when released from a greater altitude (Figure 3 and Figure 4), the proportion of moths recaptured at each distance between the release routes did not significantly differ between release heights (F_3,68_ = 3.04, *p* = 0.0858). 

The proportion of recaptured moths and their distribution in the plot varied between days after release. Of the moths that were recaptured, the greatest proportion was recaptured on the second day after release (40.4%). Recaptures were relatively high on the first (20.1%), third (10.0%), and fourth (15.5%) days after release and trailed off on the fifth (7.2%), sixth (5.2%), and seventh (1.2%) days. Moth recaptures were concentrated directly under the release route and decreased with distance from the release route. This pattern was generally similar between days after release (Figure 4), with some variation. Values of Moran’s *I* for each day after release in each plot ranged from −0.7 to 0.45. Average Moran’s *I* values for each release height and day after release were similar between most days and release heights (Table 1). Moth recaptures tended to be more significantly clustered than random (Monte Carlo test, *p* < 0.05) earlier in the week of trapping, compared to later in the week.

Wind speeds were high during the experimental releases, typical for the region and season. On the first day of release, winds were 11.9 km/h out of the north, gusting to 35.7 km/h. On the second day, winds were 20.9 km/h out of the north, gusting to 51.8 km/h. On the third day, winds were 8.9 km/h out of the south, gusting to 11.9 km/h. It was difficult to isolate the effects of wind during release flights (and the subsequent recapture period) on moth distribution, dispersal, and recapture. However, a substantial proportion of marked moths was recaptured along the southern edge of the plots on the fifth and sixth days after release (Figure 4). This pattern was more pronounced when moths were released from greater altitudes. This observation is consistent with the hypothesis that moths were blown out of the experimental plots by strong northerly winds during releases and were then returning to the nearest edge of the plot later in the week. 

The east and west plots were separated by a 25 m-wide strip of orchard. Relatively few moths were recaptured in the rows of traps on either side of the border between the east and west plots. On the first day after release, 10 moths, 0.15% of all recaptured moths, were collected in the rows of traps along the border. After 7 days, this number increased to 274, 4.2% of all recaptured moths. This suggests that it is unlikely that moths released over one plot drifted into the neighboring plot. While some moths may have moved between plots on subsequent days after release, the low numbers of moths captured along the borders of the plots suggest that the movement of moths between plots was limited.

### 3.2. Comparing Recapture Rates between Aerial and Ground Release Methods

**Figure 5:**
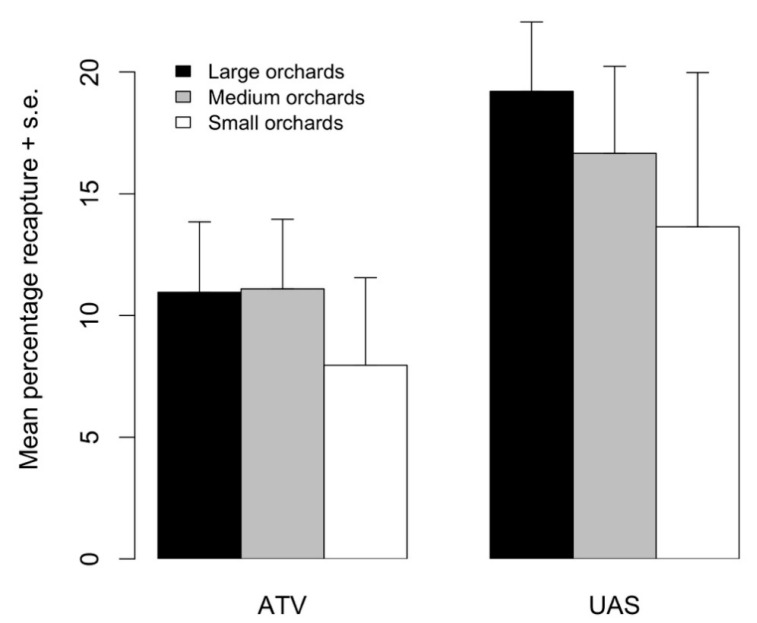
Mean percentage of moths recaptured in small, medium, and large orchards when released from an all-terrain vehicle (ATV) or uncrewed aerial system (UAS). Height of bars indicates the average percentage recapture (+1 standard error) from four replicated releases.

Overall, 10,711 marked moths were recaptured during the 2017 tests when comparing aerial and ground release methods. Total recaptures (mean ±1 standard error) tended to be higher from aerial (18.6 ± 2.50%) than ground (10.8 ± 2.44%) releases; however, this trend was only marginally different (F_1,16_ = 4.209, *p* = 0.0551). The proportion of recaptured moths did not differ between orchard sizes (F_2,15_ = 0.674, *p* = 0.534) (Figure 5). When looking at the percentage of moths that were recaptured on a given trap interval rather than the percentage of released moths that were recaptured, we found the same pattern in daily recaptures as in Experiment 1. On the first day after release, 16.3 and 27.1% of all moths were recaptured from the ground and aerial release methods, respectively. On the fourth day after release, 60% of moths from ground releases and 51% of moths from aerial releases were recaptured. On the seventh day after release, 23.5 and 23.6% of moths were recaptured from the ground and aerial releases, respectively.

UAS and ATV performed similarly well at distributing sterile moths in orchards. Mean values of Moran’s *I* were 0.09 when released from the ground and 0.08 when released by air (Figures S3–S6). In only one instance did recaptured moths exhibit significant clustering. In this orchard, moths released from UAS (*I* = 0.238, *p* = 0.01) and ATV (*I* = 0.283, *p* = 0.01) showed similar clustering (Table 1). This orchard was L-shaped with predominantly more moths recaptured in one arm of the “L”, presumably driving this pattern (Figure S3).

A total of 1193 marked moths was recaptured in the unreplicated comparison of aerial and ground release conducted in 2016: 25.3% of moths were recaptured when aerially released, while only 17.4% of moths were recaptured when released from the ground. On the first day after release, 45.8% of all recaptured moths were collected from aerial release while only 35.4% of ground recaptured moths were collected from ground release. Subsequently, 29.0 and 46.1% were collected on the second day, 16.0 and 12.7% on the third day, 5.2 and 1.4% on the fourth day, and 3.1 and 3.9% on the fifth day from the aerial and ground releases, respectively. Less than 1% of all recaptured moths were collected on the sixth and seventh days after release.

### 3.3. Comparing Recaptures between Swath and Burst Release

Overall, 1415 marked moths were collected from swath releases and 1431 marked moths were collected from burst releases in the test blocks. The total percentage of recaptured moths did not differ (*t* = 0.043, df = 3, *p* = 0.968) between swath (16.6 ± 3.57%) and burst (14.1 ± 2.78%) releases. Moths released from bursts tended to be more clustered under the point of release, while insects released in swaths were more evenly distributed across the block (Figures S7–S10). After 7 days, mean values of Moran’s *I* were 0.26 from burst releases and 0.08 from swath releases, with more instances of significant clustering (*p* < 0.05) from burst releases (Table 2).

## 4. Discussion

Aerial release has quickly become widely accepted as the preferred method for distributing sterile insects in area-wide pest management/eradication programs [30] and was the first choice for sterile codling moth release at the inception of the OKSIR program [31]. The success of SIT programs is dependent upon delivering fit, sexually competitive sterile insects to the field. The competitiveness and fitness of a sterile insect are influenced by numerous biological and operational attributes, including but not limited to transport and release technologies [37]. Evaluating moth quality in open field trials is essential to understanding how operational changes, such as using a UAS to release sterile insects, might improve or limit a program’s effectiveness [38]. As such, it was essential to (1) conduct research on how UAS technology might be used to deliver sterile insects to an orchard and (2) compare the effectiveness of UAS technology for releasing sterile insects relative to current release methods, namely ground release with ATV, to ensure that the hard-won successes of the OKSIR program are not compromised by altering release methods.

### 4.1. Comparing Release Methods

Release methods are typically divided into three types: static ground release, moving ground release, and aerial release. Depending on the specifics of a given SIT program, different methods may be employed to release different life stages of sterile insects, usually either pupae or adults [30]. This study is not the first comparison of aerial and moving ground release methods, but it contributes to our understanding of aerial and moving ground release for sterile, adult Lepidoptera. Other studies have described aerial release methods [23,32,39,40,41,42,43] and ground release methods [18,44] and have compared the releases of different life stages concurrently with different release methods [45,46]. Static ground release, moving ground release, and aerial release have all been examined to release sterile adult codling moths in BC, Canada [28,31,47], though moving ground release has been the method employed by the OKSIR program since 1994 [8]. In this study, we found that the recapture rates of sterile codling moths were 40–70% higher when moths were released from air compared to when released from the ground (25.3 vs. 17.4% and 18.6 vs. 10.8% recaptures from aerial and ground release methods, respectively, in 2016 and 2017).

We used pheromone-baited traps and recapture rates to measure the effectiveness of different release methods. Pheromone-baited traps are frequently used to measure moth quality. Measures from this method are related to other measures of moth quality performed in the field and laboratory [18,48,49]. It should be noted, however, that capture in pheromone-baited traps is not a direct measure of sexual competitiveness and that this method can be influenced by environmental [50], biological [19], and operational factors [51] that are independent of moth quality and competitiveness. In this experiment, we assumed that aerial and ground release methods would be similarly influenced by environmental, biological, and operational factors and that greater recapture rates are indicative of some underlying superiority of one method. 

We report marginally higher recapture rates from aerial release compared to ground release. One plausible explanation for this is that the survival of aerially released moths was greater than that of ground-released moths. It is likely that some aerially released moths were intercepted by the orchard canopy as they fell. There are accounts of greater recapture rates for codling moths when released into the canopy rather than on the orchard floor [18,47], with these authors hypothesizing that increased predation and/or drowning occurs on orchard floors. Our own observation is that both the codling moth and pink bollworm (*Pectinophora gossypiella* (Saunders)) experience significant mortality when chilled moths are released onto bare ground. This explanation is consistent with differences in moth recapture on the first day of release, with similar recapture percentages between methods in the following days. Presumably, moths that escaped predation on the ground on the first day of release would have migrated to the orchard canopy [52] and would have experienced similar mortality rates as those from aerial releases on subsequent days. It should be noted that daily recaptures are reported as the percentage of total recaptures on a given day. A lower percentage of recaptured moths on a given day necessitates a corresponding higher percentage of recapture on the following days as the total recaptures must add up to 100%. Thus, while the percentage of recaptured moths tended to be higher later in the week for ground releases, the total number of recaptured moths in the traps was similar to that from aerially released moths because the overall percentage of recaptured moths tended to be higher when released from a UAS.

There were a number of limitations with the experiment comparing aerial and ground release. The replication of release in this experiment was low (*n* = 4). Greater replication would have likely shown statistically significant differences between release methods. However, the marginal significance of the *p*-value *(p* = 0.0551), the large effect size (40–70% greater recapture), and the agreement in results from two separate experiments conducted in different years support our conclusion that the differences between aerial and ground releases methods are relevant. This experiment could have been further improved had there been a simultaneous release of insects from both the air and ground. Insects destined for ground release spent 1–2 h more in cold storage than those released by a UAS. However, cold storage for 24 h was shown to have no effect on codling moth flight ability [20], so it is unlikely that this additional chilling had any significant effect on our results. Lastly, the release routes travelled by the ATV and UAV were not the same, since by their very nature these two vehicles must move differently. We attempted to overcome this by placing traps on an evenly spaced grid. Codling moth capture in pheromone traps is strongly influenced by proximity to a trap [53,54]. While a given release route may have been closer to a row of traps in one method compared to another, that same release route would have been correspondingly further from an adjacent row of traps. Presumably, these confounding differences would have been averaged out over the entire array of traps in the orchard, resulting in unbiased comparisons between methods.

### 4.2. Describing the Pattern of Recaptured Moths

Experiment 1 attempted to define some of the operational parameters for the use of a UAS by the OKSIR program, namely the altitude at which sterile codling moths should be released and how widely release routes should be spaced. These parameters have been previously explored for other SIT applications [30,32,40]; however, the release technology and release strategy must be tailored to the specific challenges of a given SIT program and the target pest [30]. The OKSIR program services a relatively small area compared to other SIT programs (ca. 3000 ha), with many small 1 ha orchards situated amongst a matrix of non-host crops and urban areas. The program releases chilled adults into pome fruit orchards and relies primarily on cultural tools to control codling moths outside commercial plantings [29]. As such, we endeavored to deploy a UAS in such a way as to meet these needs, which necessarily deviated from the established methods of other, large-scale, aerial release programs.

Previously reported altitudes of aerial release range from 30 to 750 m above ground [32,40]. It is generally accepted that insects released from a greater altitude will be more likely to drift out of the targeted release zone [32]. The swath widths reported here were comparable to another report of low level (30 m) sterile insect release [40]. There was a trend for insects to be more widely distributed when released at a greater altitude; however, the treatment groups were not significantly different. A lower release would likely produce a narrower swath; however, 25 m was the lowest altitude at which the UAS could operate and safely avoid obstacles such as powerlines, wind machines, and ornamental trees. Had we released insects from a greater altitude, we might have seen a broader distribution of insects. The measurements of swath width were also limited by the size of the test plots. Had larger test plots been used, a wider swath may have been detected. In this experiment, plot size was sacrificed to increase replication. 

The recapture rate did not differ between release heights. One might expect that insects released from high altitudes would experience greater damage when they impact the ground. Though the terminal velocity of *C. pomonella* is unknown, terminal velocities of smaller (*Aedes aegypti* = 2.5 m/s) and larger (*Ceratitis capitata* = 3–4 m/s) sterile insects have been reported [41,55]. At the range of fall speeds reported, a terminal velocity of falling codling moths would be reached in the first 10 m of descent from aerial release [46], and it is unlikely that release from any altitude above 10 m would have an increased effect on moth quality when they reached the ground. The results presented here support this finding. Bouyer et al. [41] found decreased recapture when insects were released at 100 m compared to 50 m. The difference between Bouyer et al.’s [41] findings and those presented here may be because a smaller insect, with a lower terminal velocity, was released from a greater altitude and, consequently, may have experienced greater influence due to wind and/or drifting outside the study area.

Overall, the recapture rates in these experiments (11−25%) were many times higher than those typically reported elsewhere (<1–5%) in examinations of aerial release methods [23,40,41,46,56]. However, these findings are well within the ranges of recapture reported for sterile codling moths released under different operational and environmental conditions [18]. The recapture rates in these trials are likely higher than other aerial release trials due to the small plot sizes and high trap densities.

The pattern of sterile insect distribution under the release route was similar (Figure 3) to the pattern of exponential decay reported elsewhere [40]. However, we expected sterile insects to be symmetrically distributed along the release route. More moths tended to be caught on the east side of the release route. This could have been due to environmental or operational factors. As is recommended elsewhere, releases were made either with or against the prevailing wind [55]. Unfortunately, the precision with which we measured the wind direction was low, and it is possible that releases were not made completely parallel to the prevailing wind. Flights were approximately 1.5 min long, so it is also possible that wind direction changed during releases, or that wind direction differed with altitude. The asymmetrical release pattern could also have been affected by discrepancies between different GPS programs. Orchard maps and trap locations were processed in ArcGIS while flight plans were programmed in ArduPilot. Disagreements in coordinate locations between these systems could have created a discrepancy between the planned release route over the center of the plot and the actual release route, skewing moth distribution to one side of the plot by several meters.

The pattern of recaptured insects, in both experiments, was influenced not only by how the different release methods distributed the insects but also by how the insects dispersed themselves after release. Codling moth dispersal, both within and between orchards, has been studied extensively [51,53,54,57]. Sterile male codling moths are believed to travel 200–300 m within an orchard on a given night [53] and may travel up to 10 km between orchards over their lifetime [28]. In Experiment 1, moths became less significantly clustered as the experiment progressed (Table 1). This is consistent with the notion of moth dispersal within a plot. However, trap captures tended to be clustered in the center of the plots in Experiment 1. This may be for several reasons. Firstly, this experiment used “sampling without replacement”, meaning moths that were captured close to their point of release did not have the opportunity to disperse further. Secondly, the influence of calling females moths, which were also released in the thousands, is often overlooked [51]. It is likely that the addition of thousands of calling females released into a relatively small area would influence how sterile male moths dispersed and interacted with synthetic pheromone-baited traps. By the end of the experiment, the number of moths at the edges of the plots increased. However, moths were never evenly distributed in the plots, but were randomly distributed at best according to indices of clustering. This may have consequences for the scramble competition between sterile and wild insects for wild females, and the overall effectiveness of the SIT.

### 4.3. Release Strategy

The release strategy used, burst vs. swath, had lasting effects on the distribution of recaptured moths. While data reported here and elsewhere [53] suggest that sterile codling moths disperse themselves throughout an orchard as time passes, it has also been shown that the sexual competitiveness of codling moths changes with age [58]. If the distribution of wild codling moth population is known, a targeted (burst) release may produce the best sterile/wild ratio of the youngest, most sexually competitive moths in a given area. However, if the distribution of the wild population is unknown, then a uniform release strategy (swath) may produce better sterile/wild ratios. It should be re-iterated that pheromone-baited trap captures are not a measure of direct sexual competitiveness, and that these assertions warrant further investigation. Furthermore, the number of traps in these plots was relatively small (15–25) and a smaller number of neighbors (k = 4) had to be used in this analysis of spatial autocorrelation. The effectiveness of Moran’s *I* in describing spatial autocorrelation for small sample sizes is limited, and these results should be interpreted with caution [59]. Burst releases may be suitable in treating orchards less than 2–3 ha in size or for targeting known problem areas; however, further research is warranted on this topic before making operational changes.

### 4.4. Future Research

While these experiments show that the Hermes UAS is at least as effective, if not 40–70% more effective, than current ground release methods employed by the OKSIR program, other dimensions of UAS use may need to be investigated prior to the adoption of this technology, which may vary between jurisdictions. Of particular interest would be an analysis comparing the operating costs of ATVs vs. UASs. A study of the opinions of various user groups that would be affected by the use of this technology would also be valuable before the widespread use of UAS technology is implemented. A recent study found peri-urban householders in Hastings in New Zealand were generally supportive of sterile insect release and UAS if cameras were not deployed [60].

Other interesting avenues of research could investigate how this technology might be used to increase the competitiveness of sterile insect release through the optimization of release time and temperature. Further investigations of how wind and rain might affect release from UAS are warranted. Finally, further characterization of any moth quality improvement might also be warranted, especially if there are increased costs associated with aerial release.

## 5. Conclusions

These experiments demonstrated that UAS can be used to deliver sterile codling moths to an orchard. When moths were released from 25 m above ground, 80% of the recaptured moths were collected in a 50 m wide swath beneath the release route. When moths were released into the same orchard from a greater altitude (50 m), the moths were more broadly distributed, with 67% of the recaptured moths collected in a 50 m-wide swath beneath the release route. Some evidence suggests that moths released from greater altitudes drift beyond the target area more compared to moths released at a lower altitude. Recapture rates were 40–70% higher when moths were released from the air as opposed to the ground. The differences in moth recaptures between aerial and ground releases were greatest within the first day after release, suggesting that moths from the air were intercepted by tree canopies and experienced lower mortality than those released on the ground.

## Figures and Tables

**Table 1:** Summary of Moran’s *I* values for each release height and day after release (*n* = 6). *I* values shown are the mean from all replicates, and instances of significant clustering indicate the number of replicates out of 6 where a Monte Carlo procedure identified clustering to be more significant than random (*p* < 0.05).

Day After Release	Release Height	Mean Moran’s *I*	Instances of Significant Clustering
1	25	0.11	6
	50	0.17	6
2	25	0.18	6
	50	0.17	6
3	25	0.12	3
	50	0.18	4
4	25	0.15	2
	50	0.16	3
5	25	0.13	3
	50	0.14	4
6	25	0.13	2
	50	0.14	3
7	25	0	2
	50	0	1
7 Day Total	25	0.26	6
	50	0.31	6

**Table 2:** Summary of Moran’s *I* values for each release strategy (burst or swath) collection after release (*n* = 4). *I* values Scheme 4 where a Monte Carlo procedure identified clustering to be more significant than random (*p* < 0.05).

Days After Release	Release Method	Mean Moran’s *I*	Instances of Significant Clustering
1	Burst	0.10	1
	Swath	0.06	2
4	Burst	0.23	3
	Swath	−0.06	0
7	Burst	0.19	1
	Swath	0.02	0
7 Day Total	Burst	0.26	4
	Swath	0.08	2

## Data Availability

“Experiment 1 Raw Data.xlsx” includes raw data for experiment 1, “Experiment 2 Raw Data. xlsx” and “2016 UAS and ATV Comparison Raw Data.xlsx” include raw data for experiments 2 and 3. All are available online at https://www.mdpi.com/2075-4450/12/2/159/s1.

## Supplementary Materials

The following are available online at https://www.mdpi.com/2075-4450/12/2/159/s1. Figure S1: Photograph of codling moths (*Cydia pomonella*) marked with fluorescent powder and illuminated with ultraviolet light. Both green and orange fluorescent powder can be seen in this photograph. Figure S2: Photograph showing a modified all-terrain vehicle (ATV) used to release sterile codling moths (*Cydia pomonella*) by the Okanagan–Kootenay Sterile Insect Release Program. Yellow arrow indicates location and direction of moth release. Figures S3–S6: Aerial view and schema of each replicate for experiments 2 and 3. The image shows satellite imagery of each small, medium, and large orchard block (outlined in white) in each replicate. The red and blue lines indicate the routes travelled by the ATV and UAS, respectively. The shaded circles indicate the location of pheromone traps and the percentage of moths recaptured after 7 days in each trap. The size of the circle corresponds with the percentage of moths captured. Pink circles denote traps where more moths were recaptured when released by the ATV and the light blue circles indicate traps where more moths were recaptured when released from UAS. Gray circles indicate the percentage of moths recaptured that were equal between the two release methods. Traps are spaced as close to a 50 × 50 m as possible, given each block unique dimensions. Figures S7–S10. Aerial view of the orchard blocks used in experiment 3. The shaded circles indicate the location of pheromone traps and the total numbers of moths recaptured from each UAS release strategy. The size of the circle is proportional to the total number of moths recaptured. Pink circles denote traps where more moths were recaptured when released in a burst and light blue circles indicate traps where more moths were recaptured when released in a swath. Gray circles indicate that the total number of moths recaptured were equal between the two release strategies. Traps are spaced as close to a 50 × 50 m as possible, given each block unique dimensions.

## References

[1] Food and Agriculture Organization of the United Nations, FAOSTAT. http://www.fao.org/faostat/en/#rankings/countries_by_commodity.

[2] Vreysen M.J.B., Carpenter J.E., Marec F. (2010). Improvement of the sterile insect technique for codling moth *Cydia pomonella*(Linnaeus) (Lepidoptera Tortricidae) to facilitate expansion of field application. J. Appl. Entomol..

[3] Brunner J.F., Beers E.H., Brunner J.F., Willett M.J., Warner M.J. (1993). Codling Moth. Orchard Pest Management, A Resource Book for the Pacific Northwest.

[4] Marshall J. (1951). Applied entomology in the orchards of British Columbia, 1900–1951. Proc. Entomol. Soc..

[5] Epstein L. (2014). Fifty Years Since Silent Spring. Annu. Rev. Phytopathol..

[6] Klassen W., Curtis C.F., Dyck V.A., Hendrichs J., Robinson A.S. (2005). History of the sterile insect technique. Sterile Insect Technique.

[7] Bloem S., Carpenter J.E., McCluskey A., Fugger R., Arthur S., Wood S., Vreysen M.J.B., Robinson A.S., Hendrichs J. (2007). Suppression of the codling moth *Cydia pomonella* in British Columbia, Canada using an area-wide integrated approach with an SIT component. Area-Wide Control of Insect Pests: From Research to Field Implementation.

[8] Dyck V.A., Gardiner M.G.T. (1992). Sterile-insect release program to control the codling moth *Cydia pomonella* (L.) (Lepidoptera: Olethreutidae) in British Columbia, Canada. Acta Phytopathol. Entomol. Hung..

[9] Cartier L.A. (2014). Benefit-Cost Analysis of the Okanagan-Kootenay Sterile Insect Release Program.

[10] Bloem S., Bloem K.A., Carpenter J.E., Calkins C.O. (1999). Inherited sterility in codling moth (Lepidoptera: Tortricidae): Effect of substerilizing doses of radiation on field competitiveness. Environ. Entomol..

[11] Bloem S., Bloem K.A., Carpenter J.E., Calkins C.O. (1999). Inherited sterility in codling moth (Lepidoptera: Tortricidae): Effect of substerilizing doses of radiation on insect fecundity, fertility, and control. Ann. Entomol. Soc. Am..

[12] Bloem S., Carpenter J.E., Bloem K.A., Tomlin L., Taggart S. (2004). Effect of Rearing Strategy and Gamma Radiation on Field Competitiveness of Mass-Reared Codling Moths (Lepidoptera: Tortricidae). J. Econ. Entomol..

[13] Bloem S., Bloem K.A., Carpenter J.E., Calkins C.O. (2001). Season-long releases of partially sterile males for control of codling moth (Lepidoptera: Tortricidae) in Washington apples. Environ. Entomol..

[14] Bloem S., Carpenter J.E., Dorn S. (2006). Mobility of mass-reared diapaused and nondiapaused *Cydia pomonella* (Lepidoptera: Tortricidae): Effect of different constant temperatures and lengths of cold Storage. J. Econ. Entomol..

[15] Bloem S., Carpenter J.E., Dorn S. (2006). Mobility of mass-reared diapaused and nondiapaused *Cydia pomonella* (Lepidoptera: Tortricidae): Effect of mating status and treatment with gamma radiation. J. Econ. Entomol..

[16] Chidawanyika F., Terblanche J.S. (2011). Rapid thermal responses and thermal tolerance in adult codling moth *Cydia pomonella* (Lepidoptera: Tortricidae). J. Insect Physiol..

[17] Judd G., Gardiner M. (2005). Towards eradication of codling moth in British Columbia by complimentary actions of mating disruption, tree banding and sterile insect technique: Five-year study in organic orchards. Crop Prot..

[18] Judd G.J.R., Gardiner M.G.T. (2006). Temperature, irradiation and delivery as factors affecting spring-time flight activity and recapture of mass-reared male codling moths released by the Okanagan-Kootenay sterile insect programme. J. Entomol. Soc..

[19] Judd G.J.R., Thistlewood H.M.A., Gardiner M.G.T., Lannard B.L. (2006). Is lack of mating competitiveness in spring linked to mating asynchrony between wild and mass-reared codling moths from an operational sterile insect programme?. Entomol. Exp. Appl..

[20] Matveev E., Kwon J.J., Judd G.J.R., Evenden M.L. (2017). The effect of cold storage of mass-reared codling moths (Lepidoptera: Tortricidae) on subsequent flight capacity. Can. Entomol..

[21] Taret G., Sevilla M., Wornoayporn V., Islam A., Ahmad S., Caceres C., Robinson A.S., Vreysen M.J.B. (2010). Mating compatibility among populations of codling moth *Cydia pomonella* Linnaeus (Lepidoptera: Tortricidae) from different geographic origins. J. Appl. Entomol..

[22] Blomefield T., Carpenter J.E., Vreysen M.J.B. (2011). Quality of mass-reared codling moths (Lepidoptera: Tortricidae) after long-distance transportation: 1. Logistics of shipping procedures and quality parameters as measured in the laboratory. J. Econ. Entomol..

[23] Horner R.M., Lo P.L., Rogers D.J., Walker J.T.S., Suckling D.M. (2020). Combined Effects of Mating Disruption, Insecticides, and the Sterile Insect Technique on *Cydia pomonella* in New Zealand. Insects.

[24] Howell J. (1998). Spermatophore number in codling moth *Cydia pomonella* (L.) (Lepidoptera: Olethreutidaae). Can. Entomol..

[25] Andersson M., Iwasa Y. (1996). Sexual selection. Trends Ecol. Evol..

[26] Trematerra P., Gentile P., Sciarretta A. (2004). Spatial analysis of pheromone trap catches of codling moth (Cydia pomonella) in two heterogeneous agro-ecosystems, using geostatistical techniques. Phytoparasitica.

[27] Jumean Z., Rowland E., Judd G., Gries G. (2004). Male and female *Cydia pomonella* (Lepidoptera: Olethreutidae) larvae produce and respond to aggretation pheromone. Can. Entomol..

[28] Proverbs M.D., Newton J.R., Campbell C.J. (1982). Codling moth: A pilot program of control by sterile insect release in British Columbia. Can. Entomol..

[29] Okanagan-Kootenay Sterile Insect Release Program Report or and External Review 9–13 June 2014, p. 35. https://www.oksir.org/wp-content/uploads/2016/08/OKSIR-Report-of-an-External-Review-9-13-June-2014.pdf.

[30] Dowell J., Worley J., Gomes P.J., Dyck V.A., Hendrichs J., Robinson A.S. (2005). Sterile insect supply, emergence and release. Sterile Insect Technique.

[31] Proverbs M.D., Newton J.R., Logan D.M., Brinton F.E. (1975). Codling moth control by release of radiation-sterilized moths in a pome fruit orchard and observations of other pests. J. Econ. Entomol..

[32] Tan L.T., Tan K.H. (2013). Alternative air vehicles for sterile insect technique aerial release: Alternative air vehicles for SIT aerial release. J. Appl. Entomol..

[33] Dyck V.A. (2010). Rearing Codling Moth for the Sterile Insect Technique.

[34] ArduPilot Development Team Ardupilot. https://ardupilot.org.

[35] Liebhold A.M., Elkinton J.S. (1989). Characterizing spatial patters of gypsy moth regional defoliation. For. Sci..

[36] Papadopoulos N.K., Katsoyannos B.I., Nestel D. (2003). Spatial autocorrelation analysis of a *Ceratitis capitata* (Diptera: Tephritidae) adult population in a mixed deciduous fruit orchard in northern Greece. Environ. Entomol..

[37] Calkins C.O., Parker A.G., Dyck V.A., Hendrichs J., Robinson A.S. (2005). Sterile insect quality. Sterile Insect Technique.

[38] Simmons G.S., Suckling D.M., Carpenter J.E., Addison M.F., Dyck V.A., Vreysen M.J.B. (2010). Improved quality management to Enhance the efficacy of the sterile insect technique for lepidopteran pests. J. Appl. Entomol..

[39] Andress E., Walters I., del Toro M., Shelly T. (2013). Release-recapture of sterile male mediterranean fruit flies (Diptera: Tephritidae) in Southern California. Proc. Hawaii. Entomol. Soc..

[40] Vargas R.I., Whitehand L., Walsh W.A., Spencer J.P., Hsu C.L. (1995). Aerial releases of sterile mediterranean fruit fly (diptera: Tepbritidae) by helicopter: Dispersal, recovery, and sopulation suppression. J. Econ. Entomol..

[41] Bouyer J., Culbert N.J., Dicko A.H., Pacheco M.G., Virginio J., Pedrosa M.C., Garziera L., Pinto A.T.M., Klaptocz A., Germann J. (2020). Field performance of sterile male mosquitoes released from an uncrewed aerial vehicle. Sci. Robot..

[42] Mubarqui R.L., Perez R.C., Kladt R.A., Lopez J.L.Z., Parker A., Seck M.T., Sall B., Bouyer J. (2014). The smart aerial release machine, a universal system for applying the sterile insect technique. PLoS ONE.

[43] Lo O.L., Rogers D.J., Walker J.T.S., Abbott B.H., Vandervoet T.F., Kokeny A., Horner R.M., Suckling D.M. (2020). Comparing deliveries of sterile codling moth (Lepidoptera: Tortricidae) by two types of unmanned aerial vehicle and from the ground. J. Econ. Entomol..

[44] Bjeliš M., Radunić D., Bulić P. (2013). Pre- and post-release quality of sterile *Ceratitis capitata* males released by an improved automated ground release machine: Quality of *Ceratitis capitata* released by ground release machine. J. Appl. Entomol..

[45] Cunningham R.T., Suda D., Chambers D.L., Nakagawa S. (1971). Aerial broadcast of free-falling pupae of the Mediterranean fruit fly for sterile-release programs. J. Econ. Entomol..

[46] Dominiak B.C., Campbell A.J., Worsley P., Nicol H.I. (2011). Evaluation of three ground release methods for sterile Queensland fruit fly *Bactrocera tryoni* (Froggatt) (Diptera: Tephritidae). Crop Prot..

[47] Proverbs M.D., Newton J.R., Logan D.M. (1966). Orchard assessment of the sterile male technique for control of the codling moth, *Carpocapsa pomonella* (L.) (Lepidoptera: Olethreutidae). Can. Entomol..

[48] Bloem S., Bloem K.A., Knight A.L. (1998). Assessing the quality of mass-reared codling moths (Lepidoptera: Tortricidae) by using field release-recapture tests. J. Econ. Entomol..

[49] Carpenter J.E., Blomefield T., Hight S.D. (2013). Comparison of laboratory and field bioassays of laboratory-reared *Cydia pomonella* (Lepidoptera: Tortricidae) quality and field performance. J. Appl. Entomol..

[50] Pitcairn M.J., Zalom F.G., Bentley W.J. (1990). Weather factors influencing capture of *Cydia pomonella* (Lepidoptera: Tortricidae) in pheromone traps during overwintering flight in California. Environ. Entomol..

[51] Howell J.F. (1974). The Competitive effect of field populations of codling moth on sex attractant trap efficiency. Environ. Entomol..

[52] Epstein D.L., Miller J.R., Grieshop M.J., Stelinski L.L., Gut L.J. (2011). Direct sampling of resting codling moth (Lepidoptera: Tortricidae) adults in apple tree canopies and surrounding habitats. Environ. Entomol..

[53] Adams C.G., Schenker J.H., McGhee P.S., Gut L.J., Brunner J.F., Miller J.R. (2017). Maximizing information yield from pheromone-baited monitoring traps: Estimating plume reach, trapping radius, and absolute density of *Cydia pomonella*(Lepidoptera: Tortricidae) in Michigan apple. J. Econ. Entomol..

[54] Tyson R., Thistlewood H., Judd G.J.R. (2007). Modelling dispersal of sterile male codling moths, *Cydia pomonella*, across orchard boundaries. Ecol. Model..

[55] Eyles D.K., Gardiner A., Goldsmith A. Medfly terminal velocity and implications for dispersal during air or ground release. Proceedings of the FAO/IAEA International Conference on Area-Wide Control of Insect Pests: Integrating the Sterile Insect and Related Nuclear and Other Techniques.

[56] Simmons G.S., McKemey A.R., Morrison N.I., O’Connell S., Tabashnik B.E., Claus J., Fu G., Tang G., Sledge M., Walker A.S. (2011). Field performance of a genetically engineered strain of pink bollworm. PLoS ONE.

[57] Basoalto E., Miranda M., Knight A.L., Fuentes-Contreras E. (2010). Landscape analysis of adult codling moth (Lepidoptera: Tortricidae) distribution and dispersal within typical agroecosystems dominated by apple production in central Chile. Environ. Entomol..

[58] Jones V.P., Wiman N.G. (2013). Age-based mating success in the codling moth, *Cydia pomonella*, and the obliquebanded leafroller, *Choristoneura rosaceana*. J. Insects Sci..

[59] Carrijo T.B., da Silva A.R. (2017). Modifiedn Moran’s *I* for small samples. Geogr. Anal..

[60] Paterson G., Perry G.L., Walker J.T., Suckling D.M. (2019). Peri-urban community attitudes towards codling moth trapping and suppression using the sterile insect technique in New Zealand. Insects.

