# Supplementary material for: Operational Parameters for the Aerial Release of Sterile Codling Moths Using an Uncrewed Aircraft System
Source: Insects. 2021 Feb 13;12(2):159. doi: 10.3390/insects12020159 (PMC7918654; doi:10.3390/insects12020159)
Supplement: Supplementary file 1 [file insects-12-00159-s001.zip › supplementarr--layout/insects-1085262-supple-layout.docx]

Supplementary Material: Operational Parameters for the Aerial Release of Sterile Codling Moths Using an Uncrewed Aircraft System

Evan D. Esch, Rachael M. Horner , Dustin C. Krompetz, Nathan Moses-Gonzales, Melissa R. Tesche and
David Maxwell Suckling


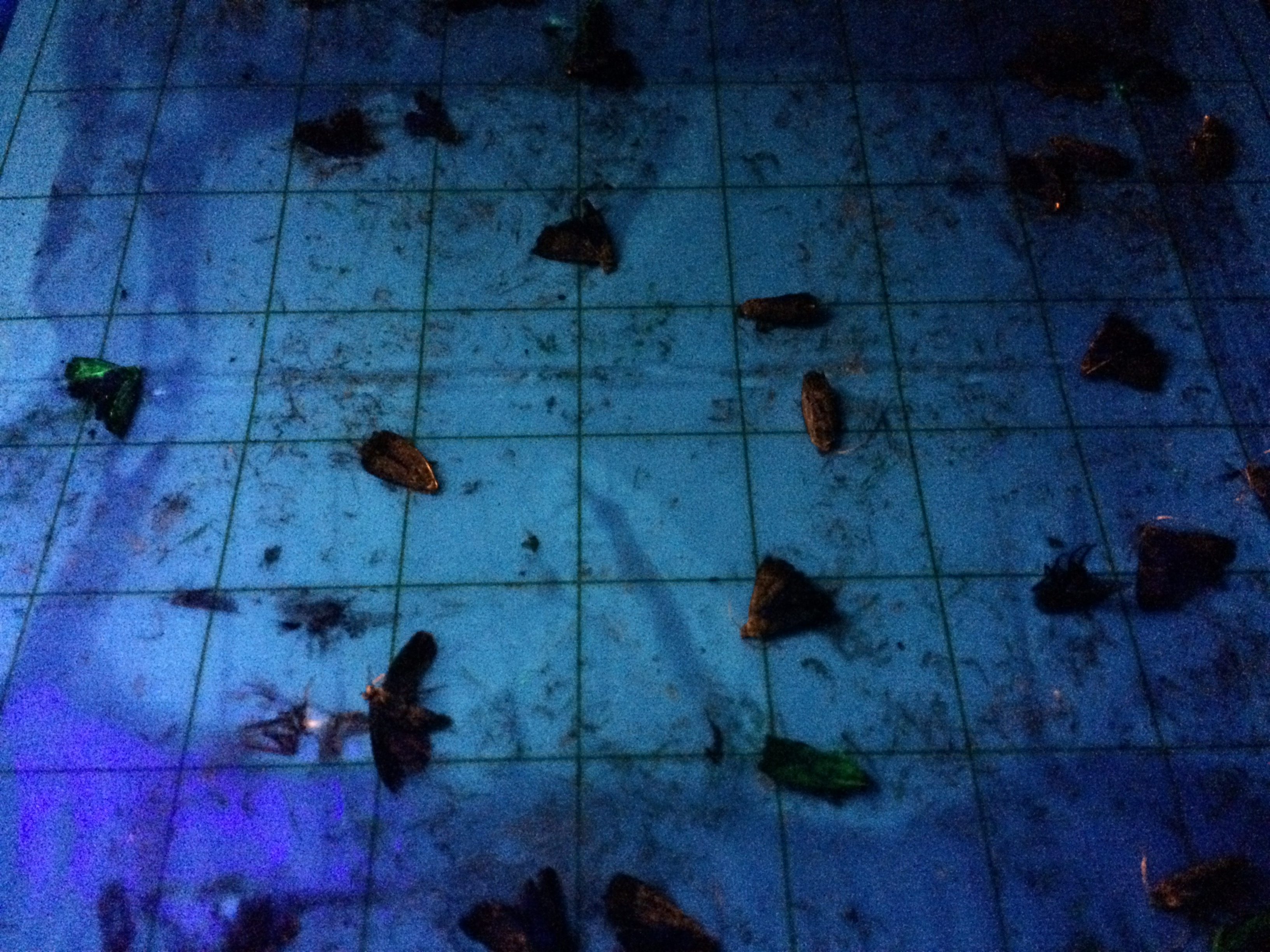


**Figure 1.** Photograph of codling moths (*Cydia pomonella*) marked with fluorescent powder and illuminated with ultraviolet light. Both green and orange fluorescent powder can be seen in this photograph.


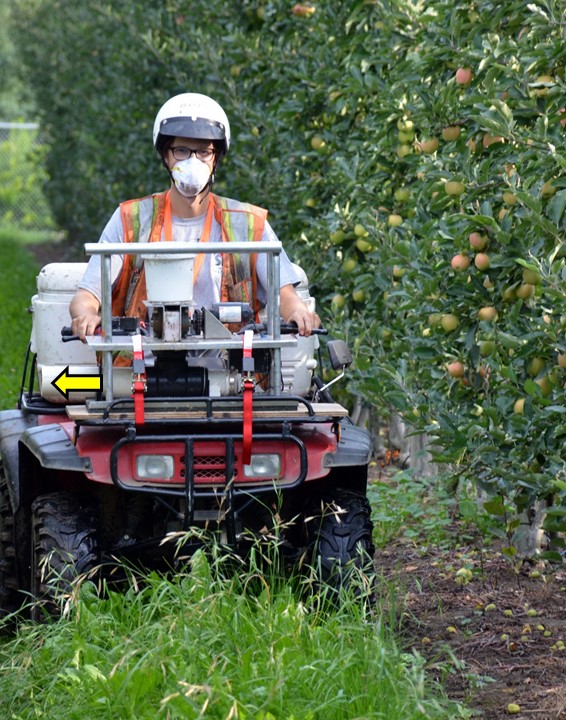


**Figure S2.** Photograph showing a modified all-terrain vehicle (ATV) used to release sterile codling moths (*Cydia pomonella*) by the Okanagan–Kootenay Sterile Insect Release Program. Yellow arrow indicates location and direction of moth release.


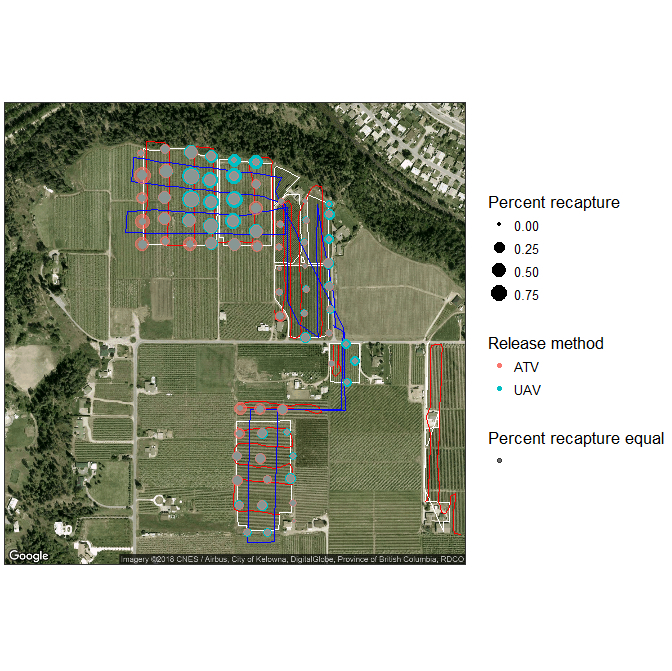


**Figure S3.** Aerial view and schema of each replicate for experiments 2 and 3. The image shows satellite imagery of each Scheme 7. days in each trap. The size of the circle corresponds with the percentage of moths captured. Pink circles denote traps where more moths were recaptured when released by the ATV and the light blue circles indicate traps where more moths were recaptured when released from UAS. Gray circles indicate the percentage of moths recaptured that were equal between the two release methods. Traps are spaced as close to a 50 × 50 m as possible, given each block unique dimensions.


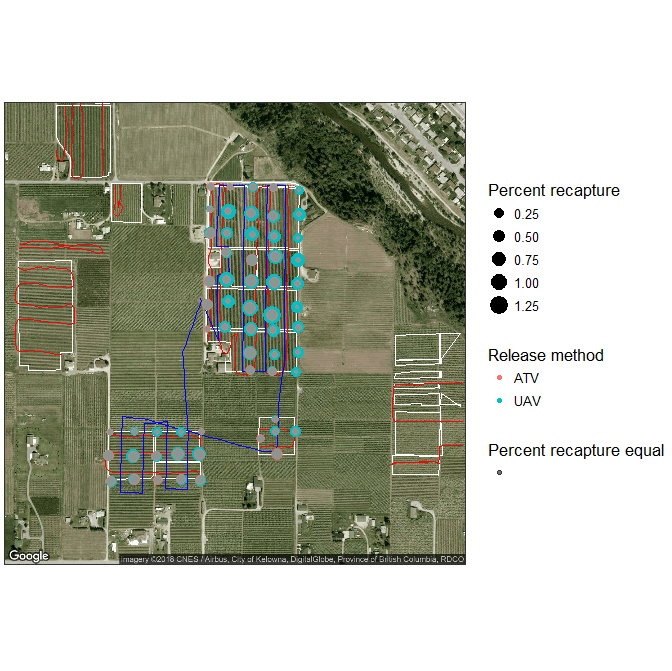
.

**Figure S4.** Aerial view and schema of each replicate for experiments 2 and 3.


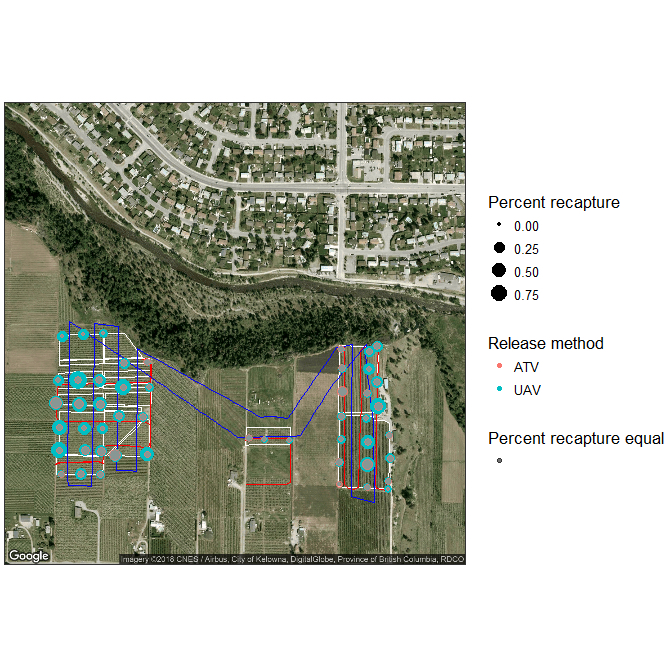


**Figure S5.** Aerial view and schema of each replicate for experiments 2 and 3..


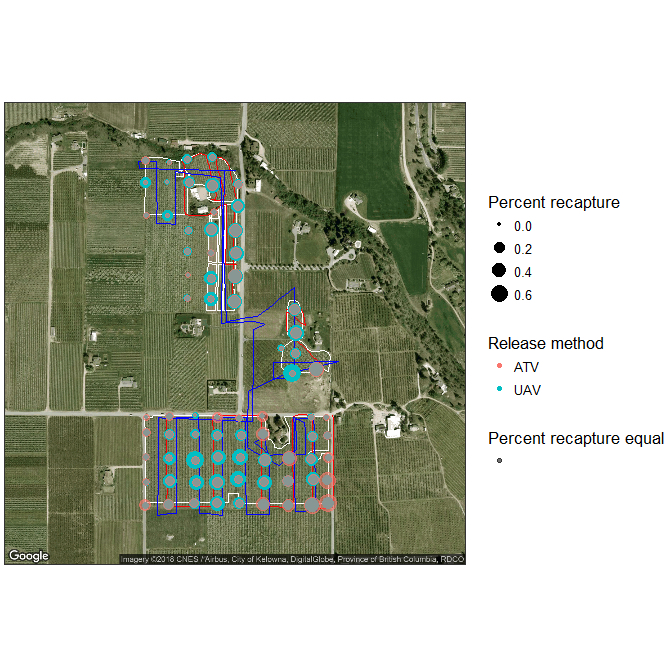


**Figure S6.** Aerial view and schema of each replicate for experiments 2 and 3.


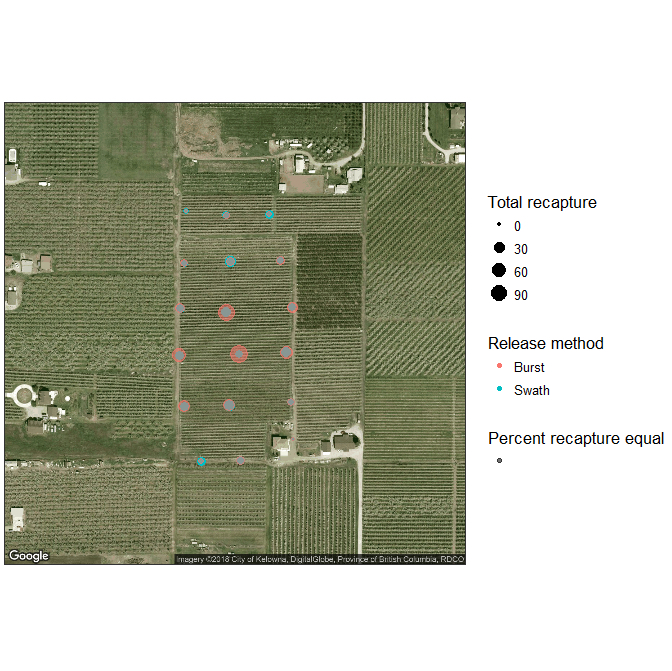


**Figure S7.** Aerial view of the orchard blocks used in experiment 3. The shaded circles indicate the location of pheromone traps and the total numbers of moths recaptured from each UAS release strategy. The size of the circle is proportional to Table 50. × 50 m as possible, given each block unique dimensions.


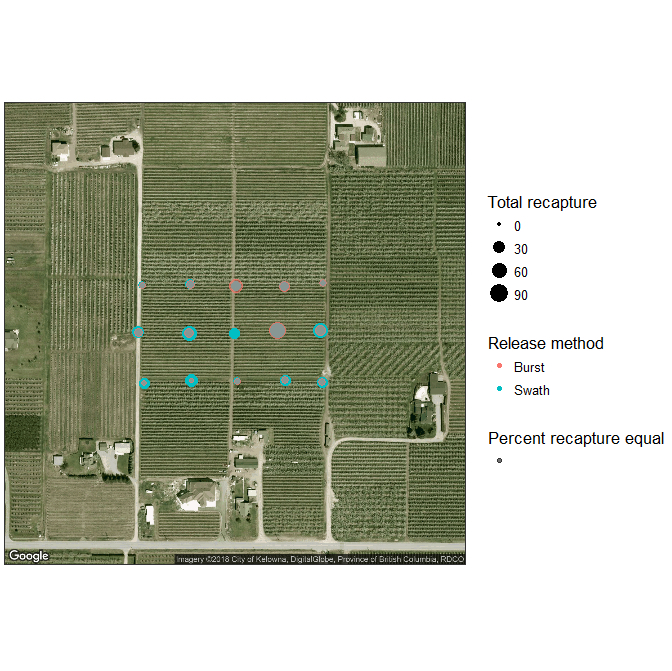


**Figure S8.** Aerial view of the orchard blocks used in experiment 3.


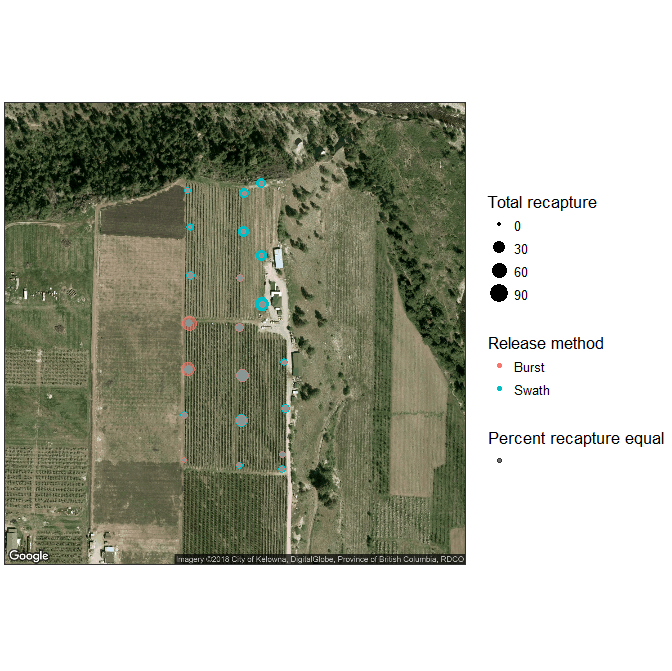


**Figure S9.** Aerial view of the orchard blocks used in experiment 3.


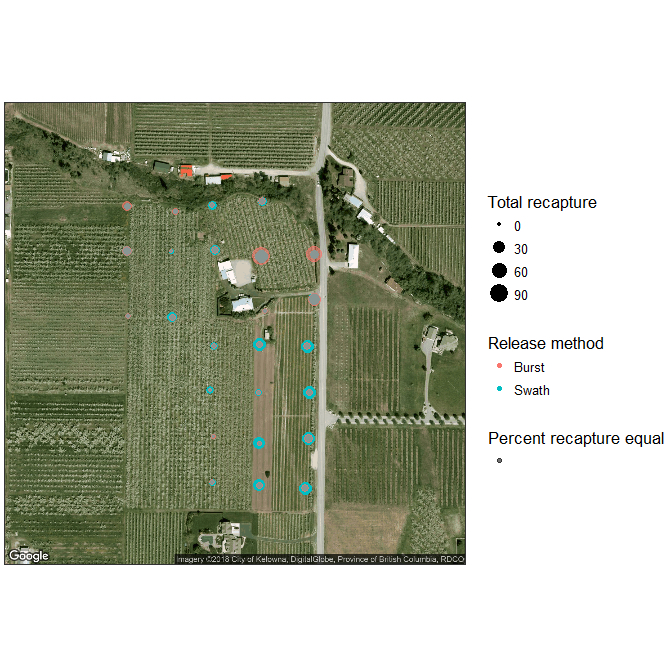


**Figure S10.** Aerial view of the orchard blocks used in experiment 3.
